# Tetramethylpyrazine Analogue T-006 Exerts Neuroprotective Effects against 6-Hydroxydopamine-Induced Parkinson's Disease *In Vitro* and *In Vivo*

**DOI:** 10.1155/2019/8169125

**Published:** 2019-11-14

**Authors:** Hefeng Zhou, Min Shao, Xuanjun Yang, Chuwen Li, Guozhen Cui, Cheng Gao, Lijun Di, Hanbing Zhong, Yuqiang Wang, Zaijun Zhang, Simon Ming-Yuen Lee

**Affiliations:** ^1^Department of Bioengineering, Zhuhai Campus of Zunyi Medical University, Zhuhai, China; ^2^State Key Laboratory of Quality Research in Chinese Medicine and Institute of Chinese Medical Sciences, University of Macau, Macau, China; ^3^Department of Biology, South University of Science and Technology, Shenzhen, China; ^4^Key Laboratory of Molecular Target & Clinical Pharmacology, School of Pharmaceutical Sciences, Guangzhou Medical University, Guangzhou, China; ^5^Cancer Center, Faculty of Health Sciences, University of Macau, Macau, China; ^6^Institute of New Drug Research, College of Pharmacy, Jinan University, Guangzhou, China

## Abstract

Parkinson's disease (PD) is a neurodegenerative disorder characterized by the progressive loss of dopaminergic neurons in the substantia nigra pars compacta (SNpc), and there is no cure for it at present. We have previously reported that the tetramethylpyrazine (TMP) derivative T-006 exhibited beneficial effects in Alzheimer's disease (AD) models. However, its effect on PD remains unclear. In the present study, we investigated the neuroprotective effects and underlying mechanisms of T-006 against 6-hydroxydopamine- (6-OHDA-) induced lesions in *in vivo* and *in vitro* PD models. Our results demonstrated that T-006 alleviated mitochondrial membrane potential loss and restored the energy metabolism and mitochondrial biogenesis that were induced by 6-OHDA in PC12 cells. In addition, animal experiments showed that administration of T-006 significantly attenuated the 6-OHDA-induced loss of tyrosine hydroxylase- (TH-) positive neurons in the SNpc, as well as dopaminergic nerve fibers in the striatum, and also increased the concentration of dopamine and its metabolites (DOPAC, HVA) in the striatum. Functional deficits were restored following T-006 treatment in 6-OHDA-lesioned mice, as demonstrated by improved motor coordination and rotational behavior. In addition, we found that the neuroprotective effects of T-006 were mediated, at least in part, by the activation of both the PKA/Akt/GSK-3*β* and CREB/PGC-1*α*/NRF-1/TFAM pathways. In summary, our findings demonstrate that T-006 could be developed as a novel neuroprotective agent for PD, and the two pathways might be promising therapeutic targets for PD.

## 1. Introduction

Parkinson's disease (PD) is the second most common neurodegenerative disorder worldwide, affecting up to 1% of the global aged population over 60 years [1]. It is mainly characterized by a progressive degeneration of dopaminergic neurons in the substantia nigra pars compacta (SNpc) [2], which leads to striatal dopamine deficiency and results in motor symptoms such as postural instability, uncontrollable tremor, rigidity, and bradykinesia [3, 4]. Apart from motor symptoms, nonmotor symptoms, including autonomic dysfunction, sleep disturbances, cognitive problems, and depression, are commonly observed in PD [5]. Current treatments for PD, including dopaminergic replacement therapy and deep brain stimulation therapy, cannot halt or slow down the progression of the disease. With the aging of the world population, PD is considered an increasing burden on society and the world economy.

Although the etiology of PD has not been fully elucidated, accumulating evidence suggests that oxidative stress, mitochondrial dysfunction, and dysregulation of glucose play a significant role in the pathogenesis of PD [6–10]. The neurotoxin 6-hydroxydopamine (6-OHDA) is widely used to produce models of PD. It has been shown that the toxicity of 6-OHDA corresponds to the increased generation of reactive oxygen species (ROS), deficient glycolytic activity, inhibition of the tricarboxylic acid (TCA) cycle, and direct inhibition of complex I and complex IV of the mitochondrial electron transport chain (ETC) [11, 12].

Glucose metabolism involves glycolysis and the pentose phosphate pathway (PPP) in the cytoplasm and the TCA cycle (Krebs cycle) and oxidative phosphorylation in mitochondria [13]. Glycolysis and the TCA cycle provide reducing equivalents for oxidative phosphorylation and finally generate ATP through the ETC in mitochondria. A decrease in glucose metabolism has been reported in PD [14]. In addition, dysfunction of the TCA cycle and oxidative phosphorylation leads to severe energy deficiency as well as increased generation of ROS in neurons [10, 15]. Moreover, several signaling pathways regulate both glucose metabolism and mitochondrial biogenesis; for example, PI3K/Akt and PGC-1*α*/NRF-1 activation was reported to exert a neuroprotective effect in PD [16–18].

**Figure 1:**
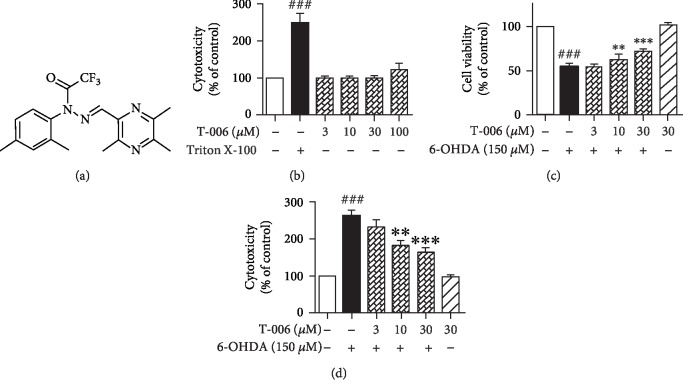
Neuroprotective effect of T-006 on 6-OHDA-induced neurotoxicity in PC12 cells. (a) Chemical structure of T-006. (b) PC12 cells were treated with different concentrations of T-006 or Triton X-100 (0.1%, *v*/*v*) for 24 h, and then cytotoxicity was analyzed by an LDH kit. PC12 cells were pretreated with T-006 (3, 10, and 30 *μ*M) or 0.1% DMSO (vehicle control) for 12 h and then challenged with 150 *μ*M 6-OHDA for an additional 24 h. Cell viability (c) and cytotoxicity (d) were measured by MTT assay and LDH assay, respectively. Data from three independent experiments are represented as the mean ± SD. ^###^*P* < 0.001 compared to the control group; ^∗∗^*P* < 0.01 and ^∗∗∗^*P* < 0.001 compared to the 6-OHDA-treated group.

We have reported that a multifunctional compound, T-006, in which the methoxyphenyl group of J147 was replaced with tetramethylpyrazine (TMP) (see Figure 1(a)), exhibited beneficial effects in Alzheimer's disease (AD) models [19, 20]. A previous study showed that T-006 could concurrently antagonize NMDA receptors and regulate the PI3K/Akt pathway, thereby stimulating neurite outgrowth in PC12 cells and potentiating nerve growth factor- (NGF-) induced neuritogenesis [19]. T-006 could rescue iodoacetic acid-induced neuronal loss, prevent oxidative stress-induced neurotoxicity, and reduce glutamate-induced excitotoxicity in cerebellar granule neurons, as well as significantly ameliorate memory impairments in APP/PS1 transgenic mice [20]. However, its effect on the PD remains unclear. In the present study, we aimed to investigate the neuroprotective potential of T-006 and the underlying mechanisms of its action against 6-OHDA-induced neurotoxicity in *in vivo* and *in vitro* models.

## 2. Materials and Methods

### 2.1. Materials

6-OHDA, dimethyl sulfoxide (DMSO), paraformaldehyde (PFA), and thiazolyl blue tetrazolium bromide (MTT) were purchased from Sigma-Aldrich (St. Louis, MO, USA). A lactate dehydrogenase (LDH) kit and cocktail were purchased from Roche Applied Science (Indianapolis, IN, USA). F-12K medium, FBS, HS, penicillin-streptomycin (PS), trypsin-EDTA, and PBS were purchased from Life Technologies (Grand Island, NY, USA). Enhanced chemiluminescence (ECL) solution was obtained from Thermo Fisher Scientific (Rockford, IL, USA). RIPA lysis buffer was bought from Beyotime Biotechnology (Shanghai, China). H-89 was purchased from Selleck Chemicals (Shanghai, China). SYBR® Premix Ex Taq™ II kit was purchased from TaKaRa. (Dalian, China). Antibodies against p-PKA, PKA, p-Akt, Akt, p-CREB, CREB, p-PI3K, PI3K, p-GSK-3*β*, GSK-3*β*, *β*-actin, lamin B1, and HRP-conjugated anti-rabbit IgG were purchased from Cell Signaling Technology (Boston, MA, USA). Antibodies against Nrf2, PGC-1*α*, NRF1, and TFAM were purchased from Santa Cruz Biotechnology (Santa Cruz, CA, USA). XF cell culture microplates and XF96 extracellular flux assay kits were purchased from Seahorse Bioscience (North Billerica, MA, USA). All other chemicals of analytical grade were purchased from local sources.

### 2.2. Cell Culture and Treatments

The rat adrenal rat pheochromocytoma PC12 cell line was purchased from the American Type Culture Collection (Manassas, VA, USA). Cells were cultured in F-12K medium supplemented with 10% (*v*/*v*) HS, 5% (*v*/*v*) FBS, and 1% (*v*/*v*) PS in a humidified atmosphere of 5% CO_2_ and 95% air at 37°C. Cells were treated with T-006 when they reached about 70% confluence. A stock solution of T-006 was dissolved in DMSO and added directly to the culture media to a final concentration of 0.1% (*v*/*v*) DMSO. The control cells were treated with DMSO only.

### 2.3. Cell Viability and Cytotoxicity Analysis

The methods were described in detail elsewhere [21]. In brief, PC12 cells were seeded in 96-well plates (8 × 10^3^ cells/well) for 24 h. After treatment with different concentrations of T-006 (ranging from 3 to 100 *μ*M) for 24 h, the supernatant was removed for LDH cytotoxicity analysis, while the remainder was used for MTT cell viability analysis. Absorbance was measured at 490 nm for LDH and 570 nm for MTT using a SpectraMax M5 (Wallace, Netherlands).

### 2.4. Evaluation of Mitochondrial Membrane Potential (*Δψ*m)

JC-1 dye was used to monitor mitochondrial integrity [22]. Briefly, PC12 cells were seeded into black 96-well plates (5 × 10^3^ cells/well). At the end of treatment, the cells were incubated in the dark with JC-1 (1 *μ*g/mL in medium) at 37°C for 15 min and then washed twice with warm medium. The *Δψ*m change was measured by flow cytometry (BD Biosciences, Franklin lakes, NJ, USA). The ratio of JC-1 red/green fluorescence intensity was calculated for the semiquantitative assessment of mitochondrial polarization states, and the value was normalized to the control group.

### 2.5. Preparation of Nuclear Extracts

Proteins in the nuclei of cells were isolated by the Nuclear and Cytoplasmic Protein Extraction Kit (Beyotime, Shanghai, China) according to the manufacturer's instructions. In brief, following treatments, PC12 cells were washed with ice-cold PBS, scraped in cold cytosol extraction buffer A with protease inhibitor cocktail, and incubated on ice for 10 min, followed by the addition of cytoplasmic protein extraction agent B. The mixture was centrifuged at 15,000 g for 5 min at 4°C, the pelleted nuclei were lysed in a nuclear protein extraction agent supplemented with protease inhibitor cocktail and centrifuged at 12,000 g for 5 min at 4°C, and the supernatant containing nuclear proteins was collected. The protein concentration of the nuclear was determined by the Bradford method. PGC-1*α* and NRF-1 levels were determined by Western blot analysis as described below.

### 2.6. Western Blot Analysis

Protein levels were examined using Western blot analysis as previously described [23]. Briefly, after appropriate treatment, the collected cells were lysed with RIPA lysis buffer. For the brain samples, tissues were homogenized in RIPA lysis buffer supplemented with protease inhibitor PMSF and cocktail as per manufacturer's instruction to extract protein. Protein concentration was measured by a BCA protein assay kit. The same amounts of protein samples were electrophoresed on SDS-polyacrylamide gel, transferred to PVDF membrane. Membranes were subsequently incubated overnight at 4°C with various primary antibodies in 5% fat-free dry milk-TBST [each antibody was diluted at 1 : 1000: phospho-PKA (Thr197), PKA, phospho-Akt (Ser473), Akt, phospho-CREB (Ser133), CREB, phospho-GSK-3*β* (Ser9), GSK-3*β*, and lamin B1, except *β*-actin (1 : 2000), Nrf2 (1 : 500), PGC-1*α* (1 : 500), NRF1 (1 : 500), and TFAM (1 : 500)]. The blots were then incubated with HRP-conjugated secondary antibody in TBST at a 1 : 5000 dilution for 1 h at room temperature. Protein bands were visualized with an enhanced chemiluminescence (ECL) kit. Blots were repeated at least three times for every condition. After development, the density of the bands was quantified by Image Lab Software (Bio-Rad, Hercules, CA, USA).

### 2.7. Analysis of mtDNA Copy Number

The copy number of mtDNA was determined by real-time quantitative PCR as previously described, with minor changes [24]. Real-time PCR with the SYBR® Premix Ex Taq™ II kit was performed on an *Mx3005P* qPCR *system* (Agilent Technologies, Santa Clara, CA, USA). The following primer sequences were used: D-loop-F, GGTTCTTACTTCAGGGCCATCA; D-loop-R, GATTAGACCCTGTACCATCGAGAT; 18s rRNA-F GCAATTATTCCCCATGAACG; 18s rRNA-R, GGCCTCACTAAACCATCCAA. Relative mtDNA copy number was calculated with the 2^−*△△*Ct^ method.

### 2.8. LC-MS Analysis of Cellular Metabolites and Neurotransmitters in the Striatum

Intracellular metabolites in glycolysis and tricarboxylic acid cycle were analyzed by an Agilent 1200 series HPLC system coupled with an Applied Biosystems/MDS Sciex 4000 QTRAP mass spectrometer [25]. In brief, PC12 cells were seeded in 6-well plates (5 × 10^5^ cells/well). After treatment with T-006 (30 *μ*M) for 12 h, cells were washed once with PBS and lysed in 80% (vol/vol) methanol at -78°C to extract intracellular polar metabolites. The homogenate was mixed with 200 *μ*L of ddH_2_O and centrifuged at 15,000 g for 15 min at 4°C. The supernatants were ultrafiltered using a 5 kDa ultrafiltration membrane to remove the proteins. The filtrate was lyophilized, dissolved in 50 *μ*L of ddH_2_O, and analyzed using LC-MS.

After the evaluation of animal behavioral tests, mice (15 mice per group) were killed by exposure to carbon dioxide and striata were then quickly dissected on ice. Each striatum was weighed and homogenized in 100 *μ*L ddH_2_O. Lysates were centrifuged at 15,000 g for 15 min at 4°C. The supernatant (80 *μ*L) was added with 160 *μ*L acetonitrile, vortex, centrifuged with 15000 rpm for 5 min at 4°C. Around 200 *μ*L supernatant was collected for further LC-MS analysis [26].

In dopamine LC-MS analysis, 100 ppb of 3,4-dihydroxybenzylamine hydrobromide (DHBA) was spiked into each sample as the internal standard. 10 *μ*L of the sample was injected onto a reversed-phase column (Synergi™ 4 *μ*m Polar-RP 80 Å, 150 × 4.6 mm) with an Agilent 1200 series HPLC system. The sample injected was analyzed with a gradient of mobile phase consisting of 0.1% formic acid in water (solvent A) and acetonitrile (solvent B) in 200 *μ*L/min, respectively. The details of the gradient profile were as follows: 0-10 min, 50-100%B; 10-15 min, 100%B; 15-15.1 min, 100-50%B; and 15.1-30 min, 50%B. The LC-MS data is collected by a QTRAP 4000 system operating positive MRM mode. The ion source parameters were as follows: capillary voltage 5500 V; source temperature 150°C; curtain gas 20 psi; GS1 30 psi; and GS2 30 psi.

In the LC-MS analysis for homovanillic acid (HVA) and dihyrophenylacetic acid (DHPA), 1 ppb of acephate (ACE) was spiked into each sample as the internal standard. The gradient profile used in the analysis was similar to that in the dopamine analysis, except solvents A and B which were replaced with water (solvent C) and acetonitrile (solvent D), respectively. The LC-MS data is collected by the same mass spectrometer system operating in negative MRM mode. The ion source parameters were similar as in the dopamine analysis, except the capillary voltage which became -4500 V.

### 2.9. Oxygen Consumption Rate (OCR) and Extracellular Acidification Rate (ECAR) Measurements

OCR and ECAR were measured with the XF extracellular flux analyzer (Seahorse Bioscience) as previously described [27]. In brief, PC12 cells (2 × 10^4^ cells/well) were seeded into Seahorse XF24 microplates and incubated in a humidified 5% CO_2_ atmosphere for 24 h. On the day of the experiment, the medium was replaced with Seahorse base medium and incubated in a CO_2_-free 37°C incubator for 1 h. For measuring OCR, 1 *μ*M oligomycin A, 1 *μ*M carbonyl cyanide-4-(trifluoromethoxy) phenylhydrazone (FCCP), and 1 *μ*M rotenone+1 *μ*M antimycin A were loading sequentially whereas, for ECAR, 25 mM glucose, 1 *μ*M oligomycin A, and 100 *μ*M 2-DG were applied. After finishing the XF assay, the cells were lysed with RIPA buffer (200 *μ*L/well) and the protein concentration was measured by BCA assay. The OCR and ECAR rates were normalized to the protein content and presented as pmols/min/mg protein and mpH/min/mg protein, respectively.

### 2.10. Animals and Treatment

Eight-week-old male C57BL/6 mice (purchased from the Medical Experimental Animal Center of Guangdong, China) were housed in standard cages with free access to food and water at 25°C following a 12 h:12 h light/dark cycle. All animal experiments were approved by the Research Ethics Committee of Institute of Chinese Medical Sciences, University of Macau.

Animals were randomly divided into 4 groups (15 mice per group): control group, sham group (injected with saline solution and oral gavage with olive oil), lesion group (injected with 6-OHDA and gavaged with olive oil), and T-006-treated lesion group (injected with 6-OHDA and gavaged with T-006).

The surgery was mostly based on unilateral stereotaxic injection of 6-OHDA. Mice were deeply anesthetized by 1% pentobarbital sodium (50 mg/kg) and placed in a stereotaxic frame. A total amount of 10 *μ*g 6-OHDA (5 *μ*g/*μ*L, dissolved in 0.9% NaCl/0.02% ascorbic acid) was stereotaxically injected into the right SNpc [28] (coordinates relative to AP, -3.1 mm; ML, +1.2 mm; and DV, -4.2 mm from the skull) at an injection rate of 0.5 *μ*L/min. After injection, the needle was left in place for 5 min before removal. The sham group was stereotaxically injected with the same volume (2 *μ*L) of 0.2% L-ascorbic acid/saline only.

**Figure 2:**
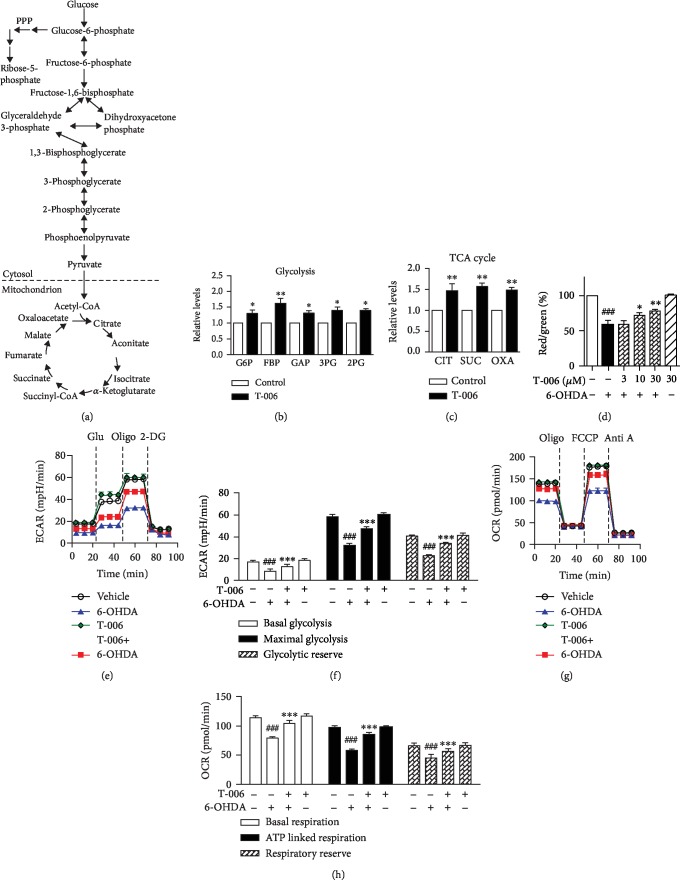
T-006 increases energy metabolism and mitochondrial functions in PC12 cells. PC12 cells were pretreated with T-006 (30 *μ*M) or 0.1% DMSO (vehicle control) for 12 h; intracellular glycolysis and TCA cycle metabolites were measured by LC-MS. (a) A simplified metabolic flow diagram of glycolysis and the TCA cycle (red, upregulated). (b) Relative levels of enhanced glycolytic intermediates. (c) Relative levels of enhanced TCA cycle intermediates. G6P: glucose 6-phosphate; FBP: fructose 1,6-phosphate; GAP: glyceraldehyde 3-phosphate; 3PG: 3-phosphoglycerate; 2PG: 2-phosphoglycerate; CIT: citrate; SUC: succinate; OXA: oxaloacetate. PC12 cells were pretreated with T-006 (30 *μ*M) or 0.1% DMSO for 12 h and then challenged with or without 150 *μ*M 6-OHDA for another 12 h. (d) PC12 cells were pretreated with T-006 (3, 10, and 30 *μ*M) or 0.1% DMSO for 12 h and then challenged with or without 150 *μ*M 6-OHDA for an additional 6 h. *Δψ*m was determined by flow cytometry. (e) The extracellular acidification rate (ECAR) was measured as an indicator of glycolytic activity using an XF24 Extracellular Flux Analyzer (normalized to protein content). The sequential injection of glucose, oligomycin, and 2-DG is indicated by vertical lines. (f) Quantitative data of basal glycolysis, maximal glycolysis, and glycolytic reserve (data from (e)). (g) The oxygen consumption rate (OCR) was measured as the mitochondrial OXPHOS activity. The sequential injection of mitochondrial inhibitors is indicated by vertical lines. (h) Quantitative data of basal respiration, ATP-linked respiration, and respiratory reserve (data from (g)). Data from three independent experiments are represented as the mean ± SD. ^###^*P* < 0.001 as compared to the control group; ^∗^*P* < 0.05, ^∗∗^*P* < 0.01, and ^∗∗∗^*P* < 0.001, as compared to the 6-OHDA-treated group.

The experimental timeline is shown in Figure 2(a). For the sham group and lesion group, mice received olive oil (0.2 ml/day) by oral gavage once daily. Mice in the T-006-treated lesion group received T-006 (3 mg/kg/day, dissolved in olive oil) by oral gavage once daily, which begin 2 days prior to 6-OHDA lesioning and continued for another 14 days. This dose of T-006 (3 mg/kg/day) was selected on the basis of previously published reports [20].

### 2.11. Behavioral Analysis

For the rotation test, mice received a subcutaneous injection of apomorphine (0.5 mg/kg) in 0.9% saline [26]. Turning behavior was monitored directly after injection for 15 min by videotaping. Each 360° turn of the body axis was manually counted as a rotation. Values were expressed as the mean of contralateral turns collected during the 5-15 min period.

For the rotarod test, the mice were trained five days to reach a stable performance, and five mice separated by large black disks were tested simultaneously. After the mice had been placed on the rotarod at a constant rotation speed of 22 rpm [26, 29], the trial was started and sustained for 10 min. The trial stopped when the mouse fell down (activating a switch that automatically stopped the timer) or when 10 min had elapsed.

### 2.12. Immunohistochemistry

Mice were transcardially perfused with PFA in PBS (pH 7.4). Brains were dissected, post-fixed in PFA overnight, and then dehydrated in 30% sucrose in PBS at 4°C. Sections were incubated in a monoclonal anti-TH primary antibody (1 : 400, Millipore; MAB318) for 48 h at 4°C, washed in PBS, and incubated with secondary anti-rabbit-HRP antibody. Immunostaining was visualized after 3-3′diaminobenzidine (DAB) staining (Vector Laboratories, Burlingame, CA, USA) using bright field microscopy (Leica, Wetzlar, Germany). Five sections through the SNc areas (-5.3 mm AP from Bregma) were randomly selected from each animal. The unbiased stereological counting of TH-positive neurons was performed bilaterally in the SNc areas. A detailed description of the stereological counting procedure is provided in supplementary materials (S1 File). The number of TH-positive neurons in the ipsilateral side and contralateral control side was calculated. Values were then expressed as % of the contralateral control side. For quantitative assessment of TH-immunoreactive fiber density in the striatum region, the optical density was measured by ImageJ software. Briefly, the background was firstly removed by subtracting values obtained from regions where no staining was observed. Values were expressed as % of the optical density of the ipsilateral side compared with that of the contralateral control side.

### 2.13. Statistical Analysis

Statistical analyses were performed with one-way ANOVA followed by Tukey's multiple comparison test (two or more groups) and Student's *t*-test (two groups) using GraphPad Prism 6.0 software (GraphPad Software Inc., San Diego, CA, USA). All experiments were performed three times and in triplicate or quadruplicate. Data are presented as the means ± SD. Significance was defined as *P* < 0.05.

## 3. Results

### 3.1. T-006 Attenuates 6-OHDA-Induced Neurotoxicity in PC12 Cells

Firstly, we investigated the cytotoxicity of T-006: PC12 cells were treated with different concentrations of T-006 for 24 h, and cell viability was subsequently determined by MTT assay. As shown in Figure 1(b), T-006, at up to 30 *μ*M, did not have any cytotoxic effects on PC12 cells, and 30 *μ*M T-006 were used in further experiments. To test the protective effects of T-006 against 6-OHDA-induced neurotoxicity, PC12 cells were pretreated with T-006 for 12 h before exposure to 150 *μ*M 6-OHDA for 24 h. The results of MTT assay showed that the viability of cells incubated with 150 *μ*M 6-OHDA for 24 h was 54.2% (Figure 1(c)), whereas pretreatment with 10 and 30 *μ*M of T-006 significantly increased cell viability to 62.6% and 71.3%, respectively, compared with the control group (100%). In addition, the neuroprotective activity of T-006 was confirmed by LDH assay. As shown in Figure 1(d), pretreatment with T-006 at 10 and 30 *μ*M for 12 h significantly decreased 6-OHDA-induced LDH leakage from 264.2% to 183.3% and 165.1%, respectively.

### 3.2. T-006 Increases Energy Metabolism and Mitochondrial Functions in PC12 Cells

The toxicity of 6-OHDA was associated with the collapse of cellular bioenergetics [30, 31]. We first defined the metabolic profiles of PC12 cells by measuring glycolytic and TCA cycle intermediates using LC-MS (Figure 2(a)). The relative levels of elevated intermediates were quantified, as shown in Figure 2(b). T-006 treatment resulted in an increase in the glycolytic pathway metabolites glucose 6-phosphate, fructose 6-phosphate, glyceraldehyde 3-phosphate, 3-phosphoglycerate, and 2-phosphoglycerate. T-006 also induced an increase in metabolites associated with the TCA cycle pathway, as evidenced by an increase in citrate, succinate, and oxaloacetate (Figure 2(c)).

To further characterize the involvement of T-006 on 6-OHDA-induced glycolysis dysfunction, we measured the ECAR under glucose starvation conditions and subsequent addition of oligomycin (Oligo) and 2-deoxyglucose (2-DG) was done in special 24-well plates equipped with oxygen sensors (Figure 2(e)). The ECAR was recorded in a glucose-free medium as basal ECAR; subsequently, the ECAR increase following the addition of glucose (Glu) can be sued to establish the glycolysis rate. Oligo (ATP synthase inhibitor) was used to stimulate maximal ECAR, effectively shifting metabolism from oxidative phosphorylation to glycolysis. The final addition of the glycolysis inhibitor 2-DG abolished the overall glycolysis. The difference between maximal and basal ECAR is considered the glycolytic reserve capacity of cells. PC12 cells treated with 6-OHDA for 12 h had lower basal glycolysis, maximal glycolysis, and glycolytic reserve capacity (Figure 2(f)) compared to the control, suggesting that 6-OHDA inhibits glycolysis activity. Pretreatments with 30 *μ*M T-006 significantly alleviate 6-OHDA-induced glycolytic rate collapse, as measured by ECAR, indicating that T-006 enhances glycolysis.

6-OHDA has also been shown to impair oxidative phosphorylation through direct inhibition of the activity of mitochondrial complexes I and IV [31–33]. To study whether T-006 prevents 6-OHDA-induced loss of mitochondrial respiratory capacity, we further assess mitochondrial function by the measuring live cell OCR. As shown in Figure 2(g), the OCR profiles, in the basal state and after the addition of oligo, FCCP, and rotenone plus antimycin A (Anti A), were determined in real time. Following the injection of oligo, basal OCR is expected to drop and the extent of the drop can be used to deduce ATP production (ATP linked respiration). Next, cells were exposed to FCCP, which is an uncoupler of mitochondrial oxidative phosphorylation that dissipates the transport of electrons from ATP production, resulting in maximal respiration. The difference between basal and maximal OCR is the reserve capacity. Finally, inhibitors of complex I and III were added to shut down the electron transfer, allowing the calculation of the nonmitochondrial respiration. 6-OHDA caused a decrease in basal respiration, ATP linked respiration, and respiratory reserve capacity (Figure 2(h)) compared to the control. T-006 prevented the mitochondrial OCR impairments induced by 6-OHDA and by itself also improved the basal OCR, ATP-linked OCR, and reserve capacity. This result revealed that most of the 6-OHDA-induced impairment in glycolysis and oxidative phosphorylation was abolished by T-006 pretreatment. Finally, mitochondrial function were measured using JC-1 staining. As indicated in Figure 2(d), treatment with 6-OHDA significantly decreased intracellular Δ*ψ*m to 58.8% of the control cell value, while pretreatment with T-006 markedly restored 6-OHDA-mediated intracellular Δ*ψ*m to 77.9% of the control value.

Taken together, these findings suggest that T-006 could protect against 6-OHDA-induced neurotoxicity partly via increasing glycolysis and mitochondrial respiratory function in PC12 cells.

### 3.3. T-006 Increases Glycolysis, TCA Cycle, and Mitochondrial Respiration through the PKA/Akt/GSK-3*β* Signaling Pathway

**Figure 3:**
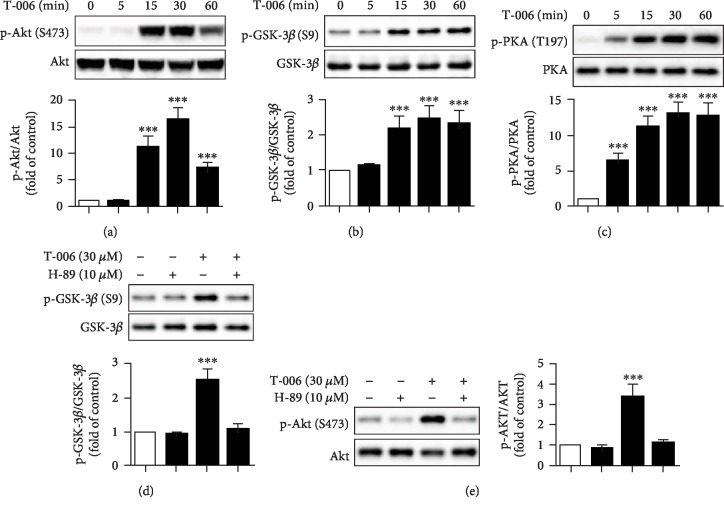
T-006 increases glycolysis, the TCA cycle, and mitochondrial respiration through the PKA/Akt/GSK-3*β* signaling pathway. PC12 cells were treated with T-006 (30 *μ*M) as indicated, and the expression ratios of phosphorylated Akt/total Akt (a), p-GSK-3*β*/total GSK-3*β* (b), and p-PKA/total PKA (c) were detected by Western blot analysis. Cells were pretreated with or without PKA inhibitor H-89 (10 *μ*M) for 1 h before exposure to T-006 (30 *μ*M) for 30 min. The expression ratios of p-GSK-3*β*/total GSK-3*β* (d) and p-Akt/total Akt (e) were detected by Western blot analysis (densitometry readings are shown at the bottom of the blots). Representative Western blot and data analysis of three independent experiments are shown. Data are represented as the means ± SD. ^∗∗∗^*P* < 0.001 compared to control.

Next, we investigated the underlying molecular mechanism by which T-006 increased glycolysis, TCA cycle, and mitochondrial respiration. Activation of PI3K/Akt/GSK-3*β* signaling is known to result in enhanced glycolysis [34–37] and mitochondrial respiration [38, 39]. The effects of T-006 on this signaling pathway were studied by Western blot analysis. As shown in Figures 3(a) and 3(b), treatment with T-006 apparently increased the phosphorylation of Akt at Ser473 (active) and GSK-3*β* at Ser9 (inactive), without affecting the total level of Akt and GSK-3*β*, respectively. However, T-006 did not change the phosphorylation of PI3K (data not shown). Several groups reported that Akt could be activated by PKA [40, 41]; we found that T-006 significantly induced the phosphorylation of PKA (Figure 3(c)). To further examine whether T-006 activates Akt/GSK-3*β* through PKA, H-89 (a PKA inhibitor) was used to pretreat PC12 cells prior to T-006 stimulation. As predicted, pretreatment with H-89 reversed T-006's effects on the phosphorylation of GSK-3*β* (Figure 3(d)) and Akt (Figure 3(e)). The results indicate that Akt activation and GSK-3*β* inactivation are regulated by PKA, and T-006 enhances glycolysis, TCA cycle, and mitochondrial respiration in PC12 cells through the PKA/Akt/GSK-3*β* signaling pathway.

### 3.4. T-006 Protects PC12 Cells against 6-OHDA-Induced Neurotoxicity through Enhancing Mitochondrial Biogenesis via the PKA/CREB/PGC-1*α* Pathway

**Figure 4:**
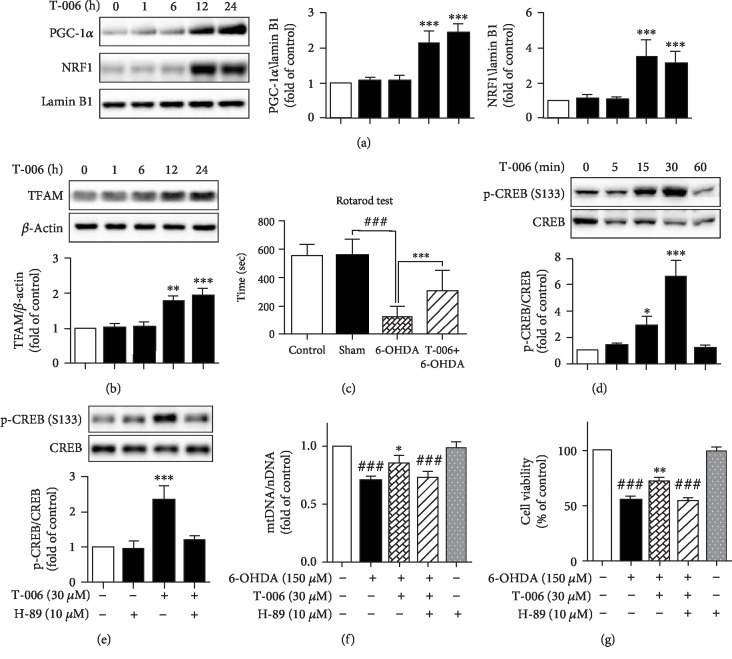
Effects of T-006 on the PKA/CREB/NRF1 signaling pathway and mitochondrial biogenesis in PC12 cells. PC12 cells were treated with T-006 (30 *μ*M) at the indicated time points, and the expression of nuclear PGC-1*α* and NRF1 (a) and cytoplasmic TFAM (b) were detected by Western blot. (c) Cells were incubated with T-006 (30 *μ*M) as indicated, and the mtDNA content was quantified by quantitative real-time PCR. (d) The cells were treated with T-006 and harvested at the indicated times; the expression of phosphorylated CREB/total CREB was detected by Western blot analysis. (e) Cells were pretreated with or without PKA inhibitor H-89 (10 *μ*M) for 1 h and then treated with T-006 (30 *μ*M) for 30 min. The expression ratios of phosphorylated CREB/total CREB were detected by Western blot. Representative Western blot and data analysis of three independent experiments are shown. Data are represented as the means ± SD. ^∗^*P* < 0.05, ^∗∗^*P* < 0.01, and ^∗∗∗^*P* < 0.001, as compared to control. PC12 cells were pretreated with T-006 (30 *μ*M) for 12 h before treatment with H-89 (10 *μ*M) for 1 h and then challenged with 150 *μ*M 6-OHDA for an additional 24 h; mtDNA content was quantified by quantitative real-time PCR (f). Cell viability was measured by MTT assay (g). Data from three independent experiments are represented as the mean ± SD. ^###^*P* < 0.001 compared to the control group; ^∗∗^*P* < 0.01 compared to the 6-OHDA-treated group.

We evaluated the effects of T-006 on mitochondrial biogenesis by investigating the expression of these three important transcriptional factors. As shown in Figure 4(a), PC12 cells were treated with T-006 for 24 h, resulting in a significant increase in the nuclear content of PGC-1*α* and its downstream target nuclear respiratory factor 1 (NRF-1). Furthermore, it was observed that the expression level of TFAM also increased significantly under these conditions (Figure 4(b)). To verify whether the elevation of nuclear PGC-1*α*, NRF1, and TFAM increases mitochondrial biogenesis, we investigated the mitochondrial DNA (mtDNA) content by real-time PCR. The results indicated that the mtDNA copy number was increased approximately 2-fold after 24 h exposure of PC12 cells to T-006 (Figure 4(c)). Furthermore, treatment with T-006 for 15 min was sufficient to enhance the phosphorylation of CREB; however, total CREB levels remained consistent (Figure 4(d)). To test whether alterations in PKA activity explained the increased CREB phosphorylation upon T-006 treatment, the cells were preincubated with 10 *μ*M PKA inhibitor H-89 for 1 h before T-006 addition, and it was shown that H-89 completely abolished the T-006-induced phosphorylation of CREB (Figure 4(e)). We also found that inhibition of PKA by H-89 blocked the increase in the mtDNA copy number (Figure 4(f)). To further confirm whether T-006 protects against 6-OHDA-induced cytotoxicity through enhancing mitochondrial biogenesis, PC12 cells were pretreated with 10 *μ*M H-89 for 1 h before T-006 addition; the results of MTT assay indicated that the protective effect of T-006 was abolished by H-89 (Figure 4(g)). Taken together, these data demonstrate that T-006 protects PC12 cells against 6-OHDA-induced neurotoxicity through enhancing mitochondrial biogenesis via the PKA/CREB/PGC-1*α* pathway.

### 3.5. T-006 Protects Mice from 6-OHDA-Induced Neurotoxicity

**Figure 5:**
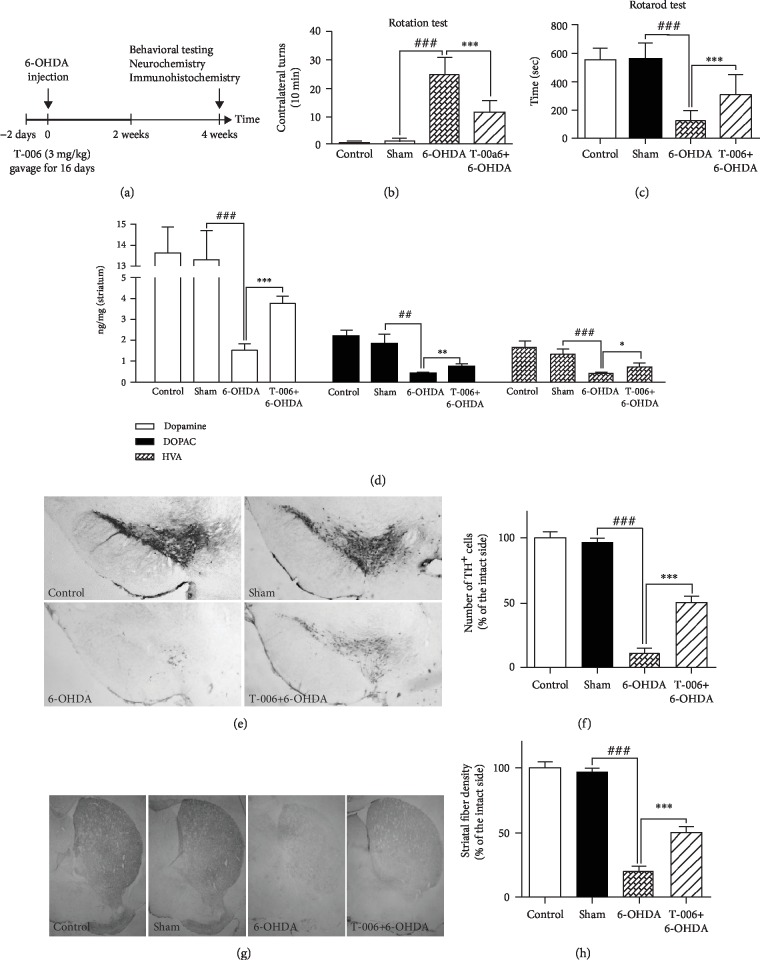
T-006 protects mice from 6-OHDA-induced neurotoxicity. (a) Experimental scheme. (b) Number of apomorphine-induced contralateral rotations (per 10 min) in mice treated with T-006 (15 mice per group). (c) For the rotarod test, the time spent on the rotating drum before falling was recorded and analyzed for each mouse (15 mice per group). (d) Concentrations of dopamine and its major metabolites (DOPAC, HVA) in the striatum were determined by LC-MS (7 mice per group). (e) Representative images of TH immunostaining in the SNpc. (f) Quantification of TH-positive cells in the SNpc (8 mice per group). (g) Representative images of TH immunostaining in the striatum. (h) Optical density of TH-positive striatal fibers (8 mice per group). Data are represented as means ± SD. ^##^*P* < 0.01 and ^###^*P* < 0.001 compared to the sham group; ^∗^*P* < 0.05, ^∗∗^*P* < 0.01, and ^∗∗∗^*P* < 0.001 compared to the 6-OHDA-treated group.

Encouraged by the cell-based results, we undertook studies to investigate whether T-006 protects mice from 6-OHDA-induced neurotoxicity *in vivo*. C57BL/6 mice were dosed orally with olive oil (0.2 ml/day) or T-006 (3 mg/kg/day) once daily. Saline (vehicle) or 6-OHDA was stereotaxically injected into the right SNpc of eight-week-old mice at day 3. The mice were treated with olive oil or T-006 for a further 2 weeks (Figure 5(a)). The results of the apomorphine-induced rotation test are shown in Figure 5(b). We observed that PD model group (6-OHDA) mice presented with a high number of apomorphine-induced contralateral rotations compared with sham group (saline-injected) mice. This behavior was significantly attenuated by treatment with T-006 in lesioned mice. For the rotarod test, the mice were trained five days to reach a stable performance; then, the time for which each mouse stayed on the rotating drum (22 rpm/min) before falling was recorded and analyzed. As shown in Figure 5(c), the 6-OHDA group exhibited a significant decrease in the time spent on the rotating drum compared with the sham group, and T-006 treatment prolonged the holding time compared with the 6-OHDA group. These results suggest that T-006 can attenuate 6-OHDA-induced motor disorder in PD mice.

After the behavioral tests, the animals were sacrificed and the concentrations of dopamine and its metabolites (DOPAC, HVA) in the lesioned striatum were analyzed. As shown in Figure 5(d), we observed that injection of 6-OHDA induced a significant decrease in the levels of dopamine, DOPAC, and HVA compared to the sham group. Interestingly, T-006 treatment significantly increased the concentrations of dopamine, DOPAC, and HVA compared with those of the 6-OHDA-lesioned mice.

We next investigated whether T-006 prevents 6-OHDA-induced degeneration of dopaminergic neurons in the SNpc *in vivo*. Immunohistochemical staining demonstrated that mice receiving saline injection presented with no significant difference in the number of TH-positive dopaminergic neurons compared to control mice (Figures 5(e) and 5(f)). The injection of 6-OHDA resulted in a marked loss of TH-positive dopaminergic neurons in the lesioned side of the SNpc. By contrast, T-006 treatment conferred significant protection against 6-OHDA-induced death of dopaminergic neurons (Figures 5(e) and 5(f)).

Furthermore, we measured the density of TH-positive dopaminergic fibers in the striatum. 6-OHDA significantly decreased striatal dopaminergic fiber density compared with saline microinjection (Figures 2(g) and 5(h)). Treatment with T-006 significantly attenuated the reduction of dopaminergic fiber density in the striatum. Altogether, these results indicate that T-006 is neuroprotective against 6-OHDA-induced toxicity in mice.

### 3.6. T-006 Treatment Activates PKA/Akt/GSK-3*β* Signaling in Brain Tissues

**Figure 6:**
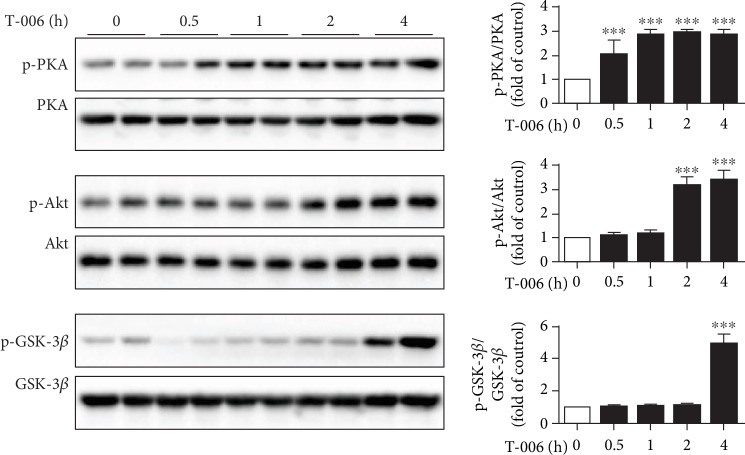
T-006 treatment activates PKA/Akt/GSK-3*β* signaling in brain tissues. C57BL/6 mice were dosed orally with either olive oil (for the 0 h time point control) or 3 mg/kg T-006 for 0.5, 1, 2, and 4 h (2 mice per group). Whole brain tissues were harvested, and the homogenates were subjected to Western blot and semiquantified by densitometry. Representative immunoblots of total PKA, Akt, GSK-3*β*, and their phosphorylated forms. Data are represented as means ± SD. ^∗∗∗^*P* < 0.001 compared to control.

Based on the above outcome, we further investigated whether T-006 could activate PKA/Akt/GSK-3*β* signaling *in vivo*. C57BL/6 mice were administered with T-006 via oral gavage at different times. The phosphorylated and total protein levels of PKA, Akt, and GSK-3*β* were analyzed by Western blot. As shown in Figure 6, we found increased phosphorylation of PKA in brain tissues after T-006 treatment for 30 min compared with control mice. Similar variations in the phosphorylation of Akt were observed following T-006 treatment for 2 h. The downstream target of Akt and GSK-3*β* was also activated 4 h after injection of T-006. These results were consistent with those obtained following T-006 treatment of PC12 cells, suggesting that the neuroprotection conferred by T-006 may be, at least in part, mediated by the activation of the PKA/Akt/GSK-3*β* signaling pathway.

## 4. Discussion

We have previously reported that the TMP derivative, T-006, exhibited a wide range of neuroprotective effects in AD models [19, 20]. In the present study, we showed for the first time that T-006 also protects against 6-OHDA-induced neurotoxicity in PD models. We further studied the molecular mechanisms underlying the protective effects of T-006 in rat pheochromocytoma (PC12) cells and C57BL/6 mice. We showed that T-006 prevented 6-OHDA-mediated depolarization of the mitochondrial membrane potential (*Δψ*m) and restored the energy metabolism and mitochondrial biogenesis in 6-OHDA-injured PC12 cells. Furthermore, T-006 significantly attenuated the loss of TH-positive neurons in the SNpc, increased the concentration of dopamine and its metabolites (DOPAC, HVA) in the striatum, and restored motor functions in unilateral 6-OHDA-lesioned mice. The neuroprotective effects of T-006 are mediated, at least in part, by the activation of both the PKA/Akt/GSK-3*β* and CREB/PGC-1*α*/NRF-1/TFAM pathways.

Mitochondria play a central role in the regulation of the cell apoptotic pathway, and a decrease in the mitochondrial membrane potential (Δ*ψ*m) has been shown to follow cell apoptosis. 6-OHDA has been widely used to generate both *in vivo* and *in vitro* models of PD [42], and the toxicity of 6-OHDA was associated with the collapse in Δ*ψ*m. Here, we showed that pretreatment of PC12 cells with T-006 could attenuate 6-OHDA-induced neurotoxicity (Figure 1). Furthermore, it was observed that T-006 prevented 6-OHDA-induced Δ*ψ*m loss, suggesting that T-006 may produce neuroprotective effects via the preservation of mitochondrial membrane integrity.

It is known that brain energy supply almost requires the oxidation of glucose through glycolysis, the TCA cycle, and oxidative phosphorylation [43, 44]. Accumulating evidence indicates that impaired energy metabolism plays a prominent role in the pathogenesis of PD [45], and the toxicity of 6-OHDA is associated with the impairment of cellular energy metabolism through the collapse of glycolytic activity and inhibition of mitochondrial complexes I and IV. Here, we showed that T-006 could directly inhibit the decrease in glycolysis rate and mitochondrial respiratory capacity caused by 6-OHDA (Figure 3). We also observed that T-006 accelerated the TCA cycle, as evidenced by an increase in the important intermediate products including citrate, succinate, and oxaloacetate (Figure 3). Akt activation can increase the rate of glycolysis by promoting the translocation of glucose transporters (Glut1 and Glut4) to the cell surface and enhancing the activity of glycolytic enzymes (hexokinase and phosphofructokinase) [46, 47]. Inhibition of GSK-3*β* (phosphorylated) by Akt resulted in the enzymatic activation of pyruvate dehydrogenase (PDH), which catalyzes the conversion of pyruvate into acetyl coenzyme A, thereby promoting the TCA cycle [47]. Moreover, Akt phosphorylates subunits *α* and *β* of ATP synthase, resulting in an increase in mitochondrial respiration [39, 48]. We further investigated the molecular mechanism by which T-006 promotes energy metabolism and showed that T-006 could activate Akt, which inhibited the activity of GSK-3*β* by increasing phosphorylation at site Ser9 (Figure 4). Furthermore, adding T-006 rapidly increased p-PKA levels, while PKA inhibitor H-89 prevented the effects of T-006 on the phosphorylation of Akt and GSK-3*β* (Figure 4), suggesting that T-006 induced GSK-3*β* inactivation via PKA signaling cascade. More interestingly, we showed that T-006 significantly activated PKA/Akt/GSK-3*β* signaling in a mouse brain (Figure 6). These data indicate that T-006-promoted energy metabolism may involve these signaling pathways.

It is generally accepted that 6-OHDA-induced neurotoxicity is mediated by mitochondrial dysfunction [12, 49–51], and mitochondrial dysfunction is a major contributor to the pathogenesis of PD [52–54]. Promotion of mitochondrial biogenesis could restore mitochondrial function and protect against mitochondrial insults in PD [17, 55, 56]. Transcriptional coactivator PGC-1*α* is a powerful regulator of mitochondrial biogenesis and cellular energy metabolism [17], and it has previously been reported that overexpression of PGC-1*α* protects against neurodegenerative disease [17, 57]. Our mechanistic studies demonstrate that T-006 could promote mitochondrial biogenesis (Figure 2) through upregulating the expression of PGC-1*α*, NRF1, and TFAM (Figure 5). In order to investigate the mechanisms underlying T-006-mediated promotion of mitochondrial biogenesis, we first analyzed the phosphorylation status of CREB, a transcription factor inducing PGC-1*α* expression. We showed that T-006 significantly activated CREB within a short timeframe (i.e., within 15 min) (Figure 2). Furthermore, the expression of *PGC*-1*α* and its downstream gene is under the control of the PKA-CREB pathway (Figure 5). Our results were in agreement with other studies showing that PKA is an upstream factor regulating CREB [58]. We showed that T-006 increases phospho-CREB which could be completely abolished by PKA inhibitor H-89 (Figure 5). What is more, pretreatment with H-89 blocked mitochondrial biogenesis and attenuated the neuroprotection exerted by T-006 (Figure 5). These data demonstrate that T-006 protects PC12 cells against 6-OHDA-induced neurotoxicity through enhancing mitochondrial biogenesis via the PKA/CREB/PGC-1*α* pathway.

It is well known that 6-OHDA induces loss of dopaminergic neurons in the SNpc through striatal dopamine depletion, which results in motor dysfunctions [42]. In the present study, we used this model for *in vivo* studies and injury was verified by an apomorphine-induced contralateral rotation test (Figure 6). We showed that treatment with T-006 significantly abolished the apomorphine-induced ipsilateral rotations resulting from 6-OHDA lesions (Figure 2). Moreover, 6-OHDA-lesioned mice exhibited significant impairment in motor function and spent less time on the rotating drum compared to the sham group; on the other hand, T-006 improved motor function after 2 weeks of treatment (Figure 2). Behavior change is probably associated with variations in the concentrations of dopamine and its metabolites [59]. We also show that 6-OHDA injection led to a major decrease in the content of dopamine and its metabolites (DOPAC, HVA) within the striatum, consistent with previous studies [59]. However, we find that T-006 can significantly increase the content of dopamine and its metabolites compared with lesioned mice (Figure 2). The decline in dopamine levels in PD is believed to arise from the severe loss of nigrostriatal dopaminergic neurons. It has been reported that 6-OHDA oxidizes and inhibits TH, which is the rate-limiting enzyme in dopamine synthesis and is widely used as a marker for dopaminergic neurons [60]. More interestingly, we show that administration of T-006 can rescue the loss of TH-positive neurons on the lesioned side of the SNpc and attenuates the reduction of dopaminergic fiber density in the striatum (Figure 2).

## 5. Conclusion

The present study provided evidence that T-006 could significantly protect against 6-OHDA-induced neurotoxicity in PD models by promoting energy metabolism and mitochondrial biogenesis. The underlying molecular mechanism is, in part, mediated by the PKA/Akt/GSK-3*β* and PKA/CREB/PGC-1*α*/NRF-1/TFAM signaling pathways. Our results suggest that T-006 could be a promising candidate for the prevention and treatment of PD.

## Data Availability

The data used to support the findings of this study are available from the corresponding authors upon request.

## Abbreviations

6-OHDA: 6-Hydroxydopamine
DOPAC: 3,4-Dihydroxyphenylacetic acid
ETC: Electron transport chain
HVA: Homovanillic acid
LDH: Lactate dehydrogenase
OXPHOS: Oxidative phosphorylation
PD: Parkinson's disease
PMSF: Phenylmethanesulfonyl fluoride
SNpc: Substantia nigra pars compacta
TMP: Tetramethylpyrazine
TH: Tyrosine hydroxylase.

## Supplementary Materials

Supplementary Materials Supplementary Material 1: Figure R1: the 2-DG abated T-006 protective effects on 6-OHDA-induced cytotoxicity. Supplementary Material 2: histological and stereological evaluation of TH-positive neurons.
