# Supplementary material for: Tetramethylpyrazine Analogue T-006 Exerts Neuroprotective Effects against 6-Hydroxydopamine-Induced Parkinson's Disease In Vitro and In Vivo
Source: Oxid Med Cell Longev. 2019 Nov 14;2019:8169125. doi: 10.1155/2019/8169125 (PMC6885178; doi:10.1155/2019/8169125)
Supplement: Supplementary Materials — Supplementary Material 1: Figure R1: the 2-DG abated T-006 protective effects on 6-OHDA-induced cytotoxicity. Supplementary Material 2: histological and stereological evaluation of TH-positive neurons. [file 8169125.f1.docx]

Supplementary material 1

Figure R1. The 2-DG abated T-006 protective effects on 6-OHDA-induced cytotoxicity.

PC12 cells were pretreated with T-006 (30 μM) or 0.1% DMSO (as control) for 12 hours, and then incubation with 150 μM 6-OHDA or 150 μM 6-OHDA plus 5 mM 2-DG, for another 24 hours. Cell viability were measured by MTT assay. Data from five independent experiments are represented as the mean ± SD. The letter “a, b” indicates p < 0.001 between two groups.

Supplementary material 2

**Histological and stereological evaluation of TH-positive neurons**

Mice were deeply anesthetized by 1% pentobarbital sodium (50 mg/kg) and perfused with PFA in PBS (pH 7.4). Brains were then dissected, post-ﬁxed in PFA overnight. The control hemisphere was marked with a shallow cut in the dorsal midbrain to allow discrimination from the treated hemisphere. Following cryo-protection by incubation in 10%, 20% and 30% of sucrose, brains were coronally sectioned at 25 µm. In order to visualize TH positive cells, successive sections through the SN were incubated with a monoclonal anti-TH primary antibody (1:400, Millipore; MAB318) for 48 hours at 4°C, washed in PBS, and incubated with secondary anti-rabbit-HRP antibody. Immunostaining was visualized after 3-3’diaminobenzidine (DAB) staining (Vector Laboratories, Burlingame, CA, USA) using bright field microscopy (Leica, Wetzlar, Germany).

For stereological quantification, the number of TH-positive neurons was counted separately for the left and right side of the brain using a 40x objective (total magnification 400x) [1]. When a section was counted, the number of TH-positive cell was multiplied by 10 for a correction of uncounted sections and corrected for split nuclei using the Abercrombie correction factor [1, 2]. Section thickness was then detected by measuring from the top to the bottom of at least 20 representative sections/brain (100x magnification) and the average thickness was calculated. In order to analyze the DA neuronal size, we measured the largest diameter of DA neurons in the x-or y-axes in each of three sections at the rostral, intermediate and caudal boundaries of the substantia nigra pars compacta (total number of cells was approximately 200 cells/animal/axis). According to previously reported method [1], the total number of TH-positive neurons was counted by the number of TH-positive cell multiplied the value of the average thickness divided by the sum of the average thickness and DA neuronal size.

Reference

1. Smeyne RJ, Breckenridge CB, Beck M, et al. Assessment of the Effects of MPTP and Paraquat on Dopaminergic Neurons and Microglia in the Substantia Nigra Pars Compacta of C57BL/6 Mice. PloS one 2016;11:e0164094.

2. Abercrombie M. Estimation of nuclear population from microtome sections. Anat Rec 1946;94:239-247.
